# A Systematic Review of Internet-Based Worksite Wellness Approaches for Cardiovascular Disease Risk Management: Outcomes, Challenges & Opportunities

**DOI:** 10.1371/journal.pone.0083594

**Published:** 2014-01-08

**Authors:** Ehimen C. Aneni, Lara L. Roberson, Wasim Maziak, Arthur S. Agatston, Theodore Feldman, Maribeth Rouseff, Thinh H. Tran, Roger S. Blumenthal, Michael J. Blaha, Ron Blankstein, Mouaz H. Al-Mallah, Matthew J. Budoff, Khurram Nasir

**Affiliations:** 1 Center for Prevention and Wellness Research, Baptist Health Medical Group, Miami Beach, Florida, United States of America; 2 Baptist Health South Florida, Miami, Florida, United States of America; 3 Department of Epidemiology, Robert Stempel College of Public Health, Florida International University, Miami, Florida, United States of America; 4 The Johns Hopkins Ciccarone Center for the Prevention of Heart Disease, Baltimore, Maryland, United States of America; 5 Brigham and Women's Hospital, Harvard School of Medicine, Boston, Massachusetts, United States of America; 6 King Abdul Aziz Cardiac Center, Riyadh, Saudi Arabia; 7 Los Angeles Biomedical Research Institute, Torrance, California, United States of America; 8 Department of Medicine, Herbert Wertheim College of Medicine, Florida International University, Miami, Florida, United States of America; University of South Australia, Australia

## Abstract

**Context:**

The internet is gaining popularity as a means of delivering employee-based cardiovascular (CV) wellness interventions though little is known about the cardiovascular health outcomes of these programs. In this review, we examined the effectiveness of internet-based employee cardiovascular wellness and prevention programs.

**Evidence Acquisition:**

We conducted a systematic review by searching PubMed, Web of Science and Cochrane library for all published studies on internet-based programs aimed at improving CV health among employees up to November 2012. We grouped the outcomes according to the American Heart Association (AHA) indicators of cardiovascular wellbeing – weight, BP, lipids, smoking, physical activity, diet, and blood glucose.

**Evidence Synthesis:**

A total of 18 randomized trials and 11 follow-up studies met our inclusion/exclusion criteria. Follow-up duration ranged from 6 – 24 months. There were significant differences in intervention types and number of components in each intervention. Modest improvements were observed in more than half of the studies with weight related outcomes while no improvement was seen in virtually all the studies with physical activity outcome. In general, internet-based programs were more successful if the interventions also included some physical contact and environmental modification, and if they were targeted at specific disease entities such as hypertension. Only a few of the studies were conducted in persons at-risk for CVD, none in blue-collar workers or low-income earners.

**Conclusion:**

Internet based programs hold promise for improving the cardiovascular wellness among employees however much work is required to fully understand its utility and long term impact especially in special/at-risk populations.

## Introduction

Despite advances in cardiovascular disease (CVD) prevention in the United States (US), CVD continues to be a major public health problem with the 2008 mortality data showing CVD accounting for 2,000 deaths per day [1]. The continuing burden of CVD in the US is largely driven by the high prevalence of major cardiovascular (CV) disease risk factors such as obesity, hypertension, diabetes, and cigarette smoking[1]. Therefore, CV health promotion in the US has witnessed a shift towards trying to improve CV health by reducing its risk factors [2]. The 2020 goals identified by the American Heart Association (AHA) put reduction of CVD mortality on par with improving CV health for all Americans[2]. The AHA's strategy of improving CV health rests on tackling seven major CVD determinants, namely blood pressure, physical activity (PA), total cholesterol (TC), healthy diet, healthy weight, non-smoking and blood glucose[1]. Based on 2007–2008 NHANES data, only 16% of US adults (about 12% of men) had the ideal for 5 or more of these metrics [1].

With about 59% of the entire US population currently in the work force, CVD prevention through worksite wellness programs provide an opportunity to reach many Americans that would have been hard to recruit otherwise. As such, the AHA has emphasized worksite-based CVD prevention, and the need for effective interventions to improve CV health among the working population [3]. On the other hand, the widespread availability and use of the internet has provided a unique and efficient avenue to engage an increasing number of people in health-related programs [4]. This is particularly relevant to remote workers and those with non-conventional schedules. As a result, employers are beginning to incorporate web-based approaches into their wellness programs.

The expansion of worksite-based wellness programs and use of the internet as delivery means, carries with it the need to sort out the evidence about their effectiveness. In the last two decades, the effectiveness of several internet-based CV wellness programs among employees have been studied with significant variance in the interventions, study design, duration and outcomes. Reviews of available evidence from such programs have been published [5]–[10], yet none has focused on internet-based CV wellness among workers.

In this systematic review we aim to synthesize the available evidence from internet-based CV wellness programs in order to guide the implementation and future development of such programs.

## Methods

We conducted a systematic review by searching PubMed, Web of Science and Cochrane library for all published studies on internet-based programs aimed at improving CV health among employees or workers up to November 2012. Using the advanced search feature we combined MESH terms and text word (tw) terms (MEDLINE or Cochrane) such as internet, web-based, online; worker, employee, workplace, worksite; and occupational health, health promotion, and wellness programs; and hypertension, blood pressure, dyslipidemia, smoking, body mass index. Table 1 outlines the search strategy in PubMed.

**Table 1:** PubMed Search Strategy.

Search number	Search terms/Combinations	Number of Items found
#9	#5 AND #6 AND #7 AND #8	32
#8	#3 AND #4	57172
#7	#1 AND #2	87124
#6	((((((((hypertension[MeSH Terms]) OR diabetes mellitus[MeSH Terms]) OR dyslipidemia[MeSH Terms]) OR diet modification[MeSH Terms]) OR exercise[MeSH Terms]) OR physical activity[MeSH Terms]) OR smoking cessation[MeSH Terms]) OR body mass index[MeSH Terms]) OR weight reduction[MeSH Terms])	799358
#5	((occupational health[MeSH Terms]) OR health promotion[MeSH Terms]) OR wellness program[MeSH Terms]	73728
#4	(((worksite[Text Word]) OR employee[Text Word]) OR worker[Text Word])	46693
#3	workplace[MeSH Terms]	12608
#2	internet[MeSH Terms]	46446
#1	(web-based[Text Word]) OR online[Text Word]	53952

In Web of Science we conducted our search employing similar search terms using the topic feature (TS). We also searched the bibliographies of the articles we found for relevant studies. We included only studies conducted in employee/working populations, whose interventions required accessing the internet and who reported CV measures of effect on employees that participated in these interventions. The CV outcomes were weight and weight related (including waist circumference, body mass index (BMI), skin fold thickness and body fat), physical activity measures, lipids [including any of total cholesterol, low density lipoprotein cholesterol(LDL-c), high lipoprotein cholesterol (HDL-c) and triglycerides], dietary changes, blood pressure, smoking cessation and blood glucose/HbA1c. We excluded case series, case reports, systematic or general reviews, studies with less than 6 months follow-up duration, and studies conducted in non-employee populations. We defined internet-based studies as those requiring study participants to log-on to the internet as part of the intervention. This included web-site use and/or email access. The database search was conducted by one reviewer (ECA) and the inclusion/exclusion criteria were applied by two independent reviewers (ECA, LLR). Only those studies that were agreed upon by both reviewers to meet the specified criteria were included in the final qualitative analysis. There was no formal protocol for this systematic review.

Methodological criteria as designed by Ogilvie et al. [11] (see appendix 1) were applied to the studies. This assessment has 2 components – suitability of study design and methodological quality criteria. Each study was assessed for both components. High quality studies were those that had grade A or B for study design and at least 4 of 6 methodological criteria. No studies were excluded due to poor quality.

## Results

**Figure 1:**
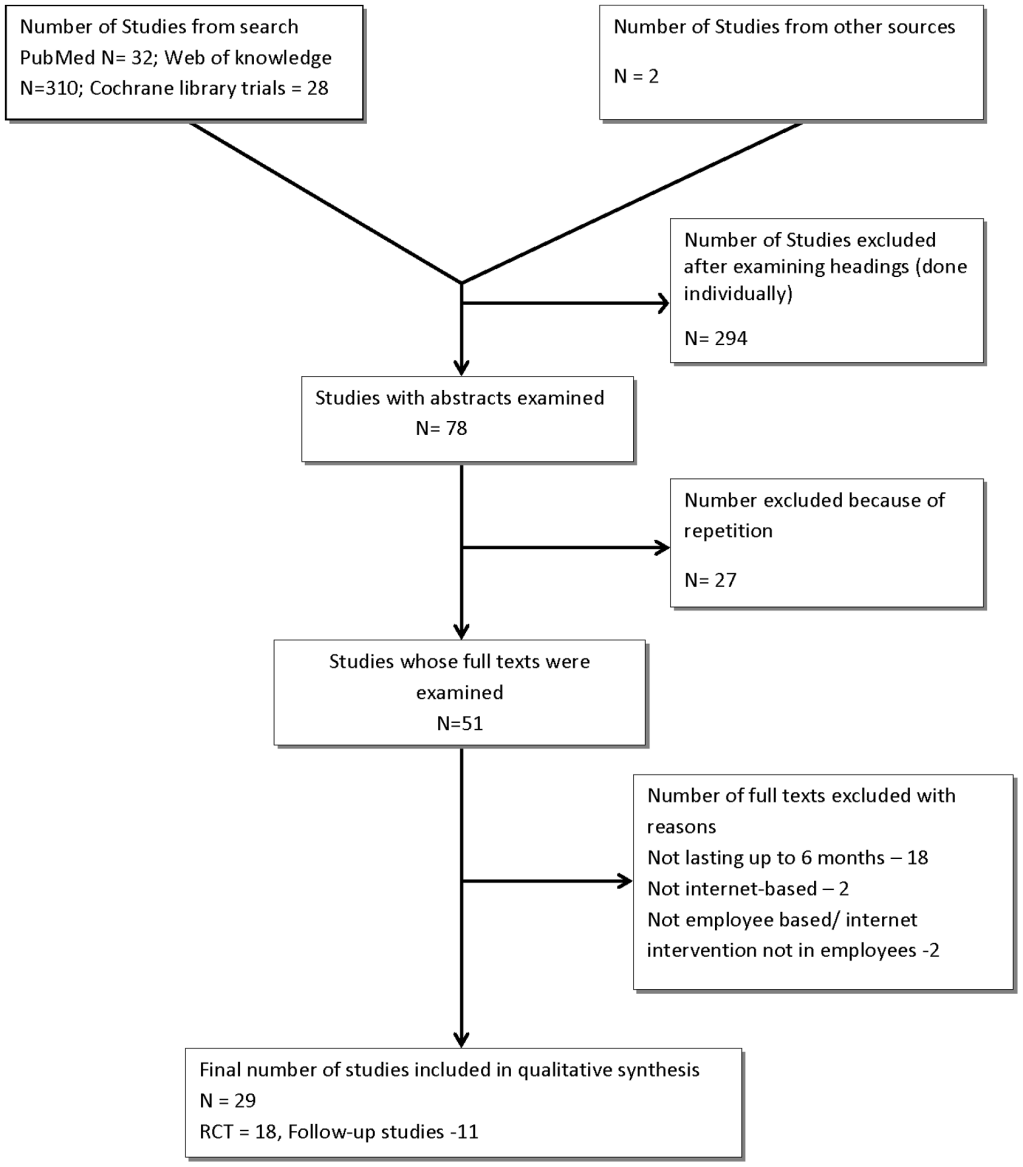
Flow Chart of Search Results.

As shown in figure 1, we included twenty-nine (29) studies in our review, 18 of which were randomized studies while the others employed pre-post design with no comparison groups. Follow-up duration was between 6 months and 2 years. We found all the randomized studies to be of high-quality. Among the non-randomized studies only one was of intermediate quality, having a comparison group and 3 of the 6 methodological quality criteria [12]. The other non-randomized studies were of low-quality. The details of the individual methodological quality scoring can be found in appendix 2.

### Description of Interventions

In general, the interventions could be grouped into largely internet-based programs with minimal interaction with the environment or study personnel (referred to as largely internet-based through out this section), or multi-component interventions in which non-internet features played more than a minor role. Among the randomized studies, 16 were largely internet-based[13]–[28], while the other 2 were multi-component studies[29], [30]. Similarly, 2 of the 10 interventions (11 studies) in the pre-post group had multiple components[31], [32], while the others were largely internet-based [12], [33]–[40]. Summary of the studies included in the review categorized according to the two main designs used can be found in tables 2 and 3.

**Table 2:** Summary of Randomized Control Trials and Quasi-Experimental Studies on Internet-Based Employee Health Programs.

Author Year	Population	Interventions	Follow- up period	Key findings
Tate 2001	Healthy overweight (BMI> = 25 – 36kg/m^2^) hospital employees Mean age 41 years IG: N = 46; CG: N = 45	**IG** - All CG interventions plus self-monitoring with e-diary; online access to therapists; weekly emails with weight loss lessons and personalized feedback; social support via e-bulletin board	6 months	**Diet & Physical activity**: NS
		**CG** - Website with basic information on weight loss, diet, exercise, other behavioral topics; 1 live counseling session; on weight control, PA.		**Anthropometrics**: *Weight loss (kg) (ΔIG vs. ΔCG)*: 4.1 vs. 1.6 p = 0.04; *WC (cm) (ΔIG vs. ΔCG)*:6.4 vs. 3.1 p = 0.009
Papadaki 2005*	Female university workers	All: Baseline dietary and psychosocial assessment	6 months	**Lipid profile (IG vs. CG)**: *ΔHDL mg/dl*: +8.5 vs. +2.3 (p = 0.036); *ΔTC: HDL*: −0.44 vs. −0.04 (p<0.01)
	Age range 25 – 55years. IG: N = 53; CG:N = 19	**IG** - Mediterranean diet website; individually tailored intervention based on baseline initial assessment **CG** - minimal feedback, print material on lifestyle changes		**Diet (IG vs. CG)**: *ΔFruits, nuts and seeds intake*: +34.9 g/d vs. −23.2 g/d; (p = 0.022).
Spittaels 2007	Healthy workers; Mean age 39.5 yrs. TA + email N = 116; TA only N = 122; CG: N = 141	**TA + email −** Online-tailored PA advice plus reinforcement e-mails. **TA only** Online-tailored PA advice only. **CG** - Online non-tailored (generic) PA advice	6 months	**Mean Body fat (%)**: *TA + email* vs. *CG*: −2.1 vs. −0.9 (p <0.05); *TA + email* vs. *TA only*: −2.1 vs. +0.1 (p<0.05)
Prochaska 2008	University employees Mean Age: 42years.	**IG**: HRA+ tailored feedback on most important improvement step (HRI) + online TTM assessments & tailored feedback +4 online tailored lifestyle intervention programs	6 months	**Physical Activity** *% with 30 minutes of moderate exercise* ≥*5 days/week*: IG vs. CG = 45.2% vs. 35.1% (p<0.01), MI group vs. CG = 46% vs. 35.1% (p<0.01);
	HRI N = 464 MI N = 433 TTM N = 503	**MI group**: HRI+ face-to-face or phone health-based MI; **CG**: HRI only		**Stress management**: IG vs. CG = 73.9% vs. 61.6% (p<0.01); MI group vs. CG = 78.2% vs. 61.6% (p<0.01)
				BMI & Smoking NS
Morgan 2009	University Males workers and students Overweight or obese IG: N = 34; CG: N = 31 Mean age = 36 years	**IG**: a face-to-face information session; Internet support through free website; dietary logs; 7 individualized emails with feedback sheets over 3 months; aimed at modification of diet and PA. **CG** -information session only	6 months	**Weight, WC, BMI, SBP, DBP, HR, PA, energy intake**: NS. Those that complied with internet use however were more likely to show reductions in weight and waist circumferences (p<0.001, p = 0.005)
van Wier 2009	Healthy overweight Mean age = 45 years	**All**: Self-help materials on overweight and healthy diet.	6 months	**Physical Activity (METmins/wk)**: ΔPG−ΔCG = 866 (p<0.05) OR = 1.8 (95%: CI 1.3; 2.6); ΔIG−ΔCG: NS.
	Phone Group N = 91 Internet Group N = 93 Control Group N = 92	**Internet group (IG)**: Educational modules on PA, diet, lifestyle; personalized counseling support; delivered through internet. **Phone group (PG)**: Similar to internet group, delivered by phone. **CG**: No intervention		**Anthropometrics** *Absolute weight loss* (Kg): ΔIG−ΔCG = −0.6 (p<0.05), ΔPG−ΔCG = −1.5 (p<0.001); *OR of* ≥*5% weight loss*: IG vs. CG = 2.3 (CI = 1.5; 3.6); PG vs. CG = 3.2 (CI: 2.1; 4.9). *Waist Circumference (cm)*: ΔIG−ΔCG = −1.2 (p<0.01); ΔPG−ΔCG = −1.9 (p<0.001).
Bennet 2011	Managerial level staff Mean age = 42 years. G 73; IG72	**IG**: 6 month web-based health and leadership program; individually tailored to the participants; **CG**: No program	6 months	**Diet** *Healthy diet attitude*: ΔIG−ΔCG = 0.25 (p<0.01);*Dietary self- efficacy*: ΔIG−ΔCG = 0.43 (p<0.01);**Anthropometrics (cm)** Δ*WC among women only*, ΔIG−ΔCG = −4.01 (p = 0.02);
van Genugten 2012	Self-reported overweight employees & general population. Mean age = 48 years IG:N = 269; CG: = 270	**IG** - Weekly online tailored lifestyle (diet, PA) education/counseling modules; online progress tracker; peer to peer forum; **CG** - Website with non-tailored information, non-tailored modules, no forums, no progress monitor	6 months	**PA Diet BMI SFT WC**: NS
Watson 2012	404 employees. Mean age = 50 years. Elevated SBP OR self-reported hypertension. IG:N = 197; CG:N = 207	**IG**: Home BP cuffs with readings transmitted to central server; access to web-site to view BP trends and read automated tailored messages; **CG**: Only access to an onsite BP machine also transmitting information to a central server.	6 months	**DBP (IG vs.CG)** *Absolute values*: 2.6 mmHg lower in IG (p<0.001); ≥*5 mmHg decline*: Overall was 27.4% vs. 15.9% (p = 0.034) Among those with hypertension was 51.5% vs. 26.4% (p = 0.01). **SBP (IG vs.CG)** *Absolute values*: No significant difference in change*% with* ≥*10 mmHg decline*: Overall was 21.3% vs. 16.4% (p = 0.044);
Slootmaker 2009	102 office workers; Mean age 32 years. IG: N = 51; CG: N = 51	**IG** - Web-based tailored PA advice based on physical activity monitor; Can set own goals; monitor activity log; lasted 3 months **CG** - Brochure with general PA advice only	8 months	**PA, aerobic fitness, body composition** NS
Papadaki 2008*	Same as Papadaki 2005	Same as Papadaki 2005	9 months	**Lipid profile (IG vs. CG)** Δ*HDL*: 0.27 vs. 0.07 (p = 0.005); Δ*TC: HDL*: −0.47 vs. −0.06 (p = 0.025) **Diet (IG vs. CG)** ΔVegetable intake g/d: 76.5 vs. 27.7(p = 0.05);
Hughes 2011	423 employees. Mean age 51 years.	**IG** - Initial web-based HRA, personalized risk profiles & tailored advice on improvement	12 months	**Physical Activity (moderate)** *IG vs. CG* = NS. *COACH vs. CG*: coefficient = 1.149 (p = 0.013);
	IG (RealAge): N = 135 COACH: N = 150 CG: N = 138	**COACH** - Initial in-person HRA+ lifestyle modification counseling; phone and email follow-up; intermittent in-person contact		**Anthropometrics (Waist circumference)** *IG vs. CG*: coefficient = −1.370 (p = .018) *COACH vs. CG*: NS
		**CG** - Printed health promotion materials		**Diet** *% energy from fat IG vs. CG* = NS; *COACH* vs. *CG*: coefficient = −2.187 (p<0.027), *Fruit and vegetable consumption IG vs. CG* = NS; *COACH vs. control* coefficient = 4.366 (p<0.001);
Thorndike 2012	Hospital staff; Post 10 week exercise program; Mean age = 43 yrs. IG:N = 174; CG:N = 154	**IG** – Web-site for goal-setting, self-monitoring of weight, exercise (pedometers) and nutrition; minimal personal contact 9 months program **CG** - No internet support	12 months	**Weight, BMI WC SBP DBP TC LDL HDL TG FPG** NS
Aittasalo 2012	241 Office workers Mean age = 45 years IG: N = 123; CG: = 118	**IG**: group counseling, pedometer, monthly email support for 6 months; targeted at increasing PA. **CG**: Only data collection	12 months	**Self-reported PA** NS
Reijonsaari 2012	Employees of insurance company Mean age: 43 years IG: 264 CG: 257	**IG** – Baseline fitness test, information leaflet on PA, accelerometer for monitoring of PA, website access for monitoring progress, received counseling over the telephone or web messages **CG** - Baseline fitness test, information leaflet on PA	12 months	**Maximal oxygen uptake, weight, WC, SBP, DBP** = Not Significant.**Body fat %** ΔIG−ΔCG = 0.6% greater increase in IG (95% CI: 0.2%–1.0%)
Kang 2010	Male industrial workers; With Type 2 DM or IFG Not treated	*Intervention*: 5 face-to-face counseling sessions on lifestyle modification (12 weeks) Follow-up emails every 3 weeks (30 weeks)	24 months	**Metabolic Profile** Δ*FPG (mmol/l) vs. CG*: *TIG* = −0.83 vs. −0.17 (p<0.05); *OIG*: −0.84 vs. −0.17 (p<0.05); Δ*HbA1c (mmol/l) vs. CG*: *TIG*: −0.15 vs. 0.27 (p<0.05); *OIG*: 0.13 vs. 0.27 (p<0.05) Δ*TC* vs. CG *TIG* = −11.12 vs. 5.75 (P = 0.05)
	OIG: N = 25, TIG: N = 25, CG: N = 75	**TIG**: Intervention in first and second year. **OIG**: Intervention only in second year		**Blood pressure (mmHg)** Δ*SBP vs. CG*: *TIG* = −10.92 vs. −0.80;
		**CG**: General health information at baseline		**Diet** Δ*Protein intake vs. control*: *TIG*: −31.63 vs. −3.99 (p<0.05) Δ*Sodium intake*: −2356.95 vs. 303.66(p<0.05) **Anthropometrics (cm)** Δ*WC vs. CG*: *TIG* = −1.76 vs. 3.9 (p<0.05)
Dekkers 2011	As described in Van Wier et al. 2009	As described in Van Wier et al. 2009	24 months	**Weight, WC, skin fold thickness, SBP, DBP, TC, maximal oxygen uptake**: No significant difference in the change between groups
Robroek 2012	924 Dutch Employees IG: N = 465 CG: N = 459	**IG**: HRA; one face-to-face counseling session Website with tailored advice based on self-reported **PA** and diet; ability to monitor progress; online support from health professionals; monthly e-mail messages for throughout year one **CG**: HRA and one face-to-face counseling session	24 months	**PA, Fruit & Vegetable intake, Obesity, SBP, DBP, TC, maximal oxygen uptake**: NS

quasi-experimental design

Δ =  change in (− reduction, + increase); MetS = Metabolic syndrome; FG = fasting glucose; WC =  waist circumference; BP =  blood pressure; SBP =  systolic blood pressure; DBP =  diastolic blood pressure; NS = not significant; TC =  total cholesterol; TG =  triglycerides; FRS = Framingham risk score; HRA = health risk assessment; HRI = health risk intervention; TTM = transtheoretical model; MI =  motivational interview; CVD = cardiovascular disease; HDL = High density lipoprotein cholesterol; lbs =  pounds; RBG = random blood glucose; IG = intervention group; CG = control group TIG  =  two year intervention group; OIG =  one year intervention group; TA =  tailored advice; PA = physical activity; N/A = not available CI = 95% confidence interval;

**Table 3:** Summary of Longitudinal/Follow-up Studies on Internet-Based Employee Health Programs.

Author, year	Study Population	Intervention and Comparisons	Follow-up period (months)	Outcomes measured and results
Jung, 2012	226 employees with ≥1 Metabolic Syndrome risk factor Mean age = 42 yrs;Subcategorized into Low risk (≤2 risk factors N = 64); High risk (>2 risk factors N = 162) All had same interventions	In-person group education, individualized workplace counseling; equipped worksite e-health zones; pedometers; individualized telephone counseling based on measured parameters; Monthly email with including BP monitoring results and pedometer step counts.	6 months	**Metabolic Profile** Δ*TG (mg/dl)*: low risk = −42.8(p = 0.0001), high risk = −35.5 (p = 0.0001), between groups = NS; Δ*HDL (mg/dl)*: low risk = NS, high risk = NS, between groups = NS Δ *FG (mg/dl)*: low-risk = 0.5, high risk = −6.3 (p = 0.848, p = 0.0001), between groups (p = 0.019)
				**Anthropometrics** Δ*WC (cm)*: low-risk = −2.7 high-risk = −2.9(p<0.001, p<0.001), between groups = NS;
				**Blood pressure (mmHg)** Δ*SBP*: low-risk = −5.2 high-risk = −5.3 (p = 0.001, p = 0.0001), between groups = NS; Δ*DBP*: low-risk = −3.3 high risk = −6.7 (p = 0.017, p<0.001), between group (p = 0.028);
Speck, 2010	619 participants in an academic worksite Mean age = N/A	Step-counting pedometer; web-site with diet information & individual e-journaling, ability to share personal step totals, motivational tips, various other health-related resources	6 months	**PA Goal** = *10,000 average daily steps* 36% of 424 met goal @ 2weeks (baseline) and 45% of 163 at follow-up and 54% of those who tracked throughout the 21 week program (N = 57). Population that tracked every week for the duration of the study = 9%
Pratt, 2006	2498 employees globally, Mean age (ranges) = 42 – 45 yrs	Website with online recipes, nutrition/fitness web-chats; online support from nutritionists & exercise specialists; motivational e-newsletters; incentives; four cohorts over 4 consecutive years	5–7 months	**Δfruit and vegetable intake** increased over the 4 years (p<0.05) **ΔPA** increased (p<0.05) **Δ weight** (yrs 1, 2 & 3) = −1.4 kg (P<0.05), **Δ weight** (yr 4) = −1.8 kg (p<0.05)
Colkesen2011	176 employees who Mean Age = 45 yrs	Web-based HRA & individually tailored adviceon healthy lifestyle (web-based health action plan); Referral for those with high CVD risk;Health counseling at request.	7 months	**Framingham risk score**Δ*FRS* (%) entire population = −4.9 (p = 0.017) high risk (FRS ≥20% @ baseline) = −17.9 (p = 0.001) **Lipid Profile (mg/dl)** Δ*TC* = +7.7 (p = 0.001); Δ*HDL* = +3.9 (p<0.001); Δ*TG* = +8.9 (p = 0.025). **Blood Pressure(mmHg)** Δ*SBP* = −5 (p<0.001) **Anthropometrics (cm)** Δ*WC* = −2 (p<0.001)
Moore, 2008	735 workers and their household members Mean age = 41 yrs	Web-site with information about healthy nutrition and tips on healthy exercise; Email reminders with link to web-site and article for the week. Online -Tailored DASH-diet based advice	12 months	**Anthropometrics** Δ*Weight (kgs)*: All weight groups = −1.41 (p<0.01) Those with BMI>25 = −1.90 (p<0.01). **Blood Pressure** Δ*SBP*: high BP group = −6.8 mmHg (p<0.001). **Diet** Δ*fruit* = increase *&* Δ *vegetables intake* = increase (p = 0.03, p = 0.002); Δ*grain intake* = decrease (p = 0.04)
Perez, 2009	214 Employees at a state DOH Mean age = NA	Online behavioral change program; wellness report after a HRA; Online progress tracking; Incentives for progress towards goals	12 months	**Vegetable intake**: Δ *in % with* ≥*3 times/d* = +12.2−(p = 0.03) **Fruit intake**: Δ *in % with* ≥*3 times/d* = +6.5 (p = 0.08)
Peterson 2008	Employees of a large multi-national company. N = 2127 Median age = NA Matched Controls N = 2127	Initial HRA; online-weight management tool; food & weight trackers; meal planners, serving size calculators; social support, dietary assistance, emails (general and personalized); Earn points for web use and progress.	12 months	**Anthropometrics** Weight *Overall* = 1.09 kg decrease over 6 months (p<0.005); *Among overweight persons* = 0.78 kg reduction over 6 months. Δ *Frequency of normal weight* (IG vs. matched controls) = +2.3% vs. +0.3%; (p<0.05)
Hotta, 2007	101 University staff; Attempting to quit smoking; Median age = 45 yrs.	5 face-to-face smoking cessation classes with personalized assessments; nicotine patches; Self-help booklet; e-mails support with motivational information; Ability to send out emails to the mailing list.	12 months	**Smoking Cessation** 50% quit rate in population using intention to treat analysis and 53% of those who completed the one year follow-up. Writing or sending emails to the mailing list was associated with continued smoking cessation for a year (OR 5.9; p = 0.008)
Graham, 2007	1772 current smokers Mean age = 44 yrs	Website with tailored smoking cessation information, help with setting quit dates, online cessation counselors & social support	12 months	**Smoking Cessation** - *7-day PPA* 12.8% in the entire sample recruited at baseline and 43% in those that adhered to the program
Sarna, 2009	246 Nurses; smoker (baseline); Mean age = 45 yrs	Website with tailored smoking cessation information, help with setting quit dates, online cessation counselors, online social support	12 months	**Smoking Cessation** - *7-day PPA* 43% of follow-up responders at 3 months; 45% at 6months; 53% at 1 year
McHugh, 2012	238 employees, BMI>25, IG N = 101; CG N = 137	**IG**: Web-site + online-tailored advice based web-based HRA; set goals, monitor diet and PA online; Email with links to monthly tailored e-newsletters **CG**: Did not participate in the program	24 months	**Blood pressure (mmHg)** Increase in the mean SBP (+3.772 p = 0.013); **Metabolic Profile** Reduction in HDL mg/dl (−7.02; p = 0.033); Increase in RBG (+3.72; p = 0.028); **BMI**: No between group differences reported

Δ =  change in (− reduction, + increase); FG = fasting glucose; WC  =  waist circumference; BP  =  blood pressure; SBP  =  systolic blood pressure; DBP  =  diastolic blood pressure; NS = not significant; TC  =  total cholesterol; TG  =  triglycerides; FRS = Framingham risk score; HRA = health risk assessment; HRI = health risk intervention; TTM = transtheoretical model; MI =  motivational interview; CVD = cardiovascular disease; HDL = High density lipoprotein cholesterol; RBG = random blood glucose; IG = intervention group; CG = control group; TA  =  tailored advice; PA = physical activity; N/A = not available; CI = 95% confidence interval; PPA = point prevalence of abstinence.

Common themes with the largely internet-based studies were provision of access to a web-site and a needs assessment either through questionnaires (health risk assessments, psychosocial assessments, health surveys, etc.) or through monitoring devices such as physical activity monitors or pedometers. Some interventions in the randomized trials also included the ability to self-monitor progress, email support for reminders or motivational messages, and social networking (interaction with others in the intervention). In general there was no clear pattern for the relation between the number of intervention components and the outcome among the internet-based randomized trials.

Multi-component studies appeared to be more effective, as all 4 studies (2 trials and 2 pre-post studies) found significant associations between their intervention and outcomes[29]–[32].

### Effects on individual outcomes

**Figure 2:**
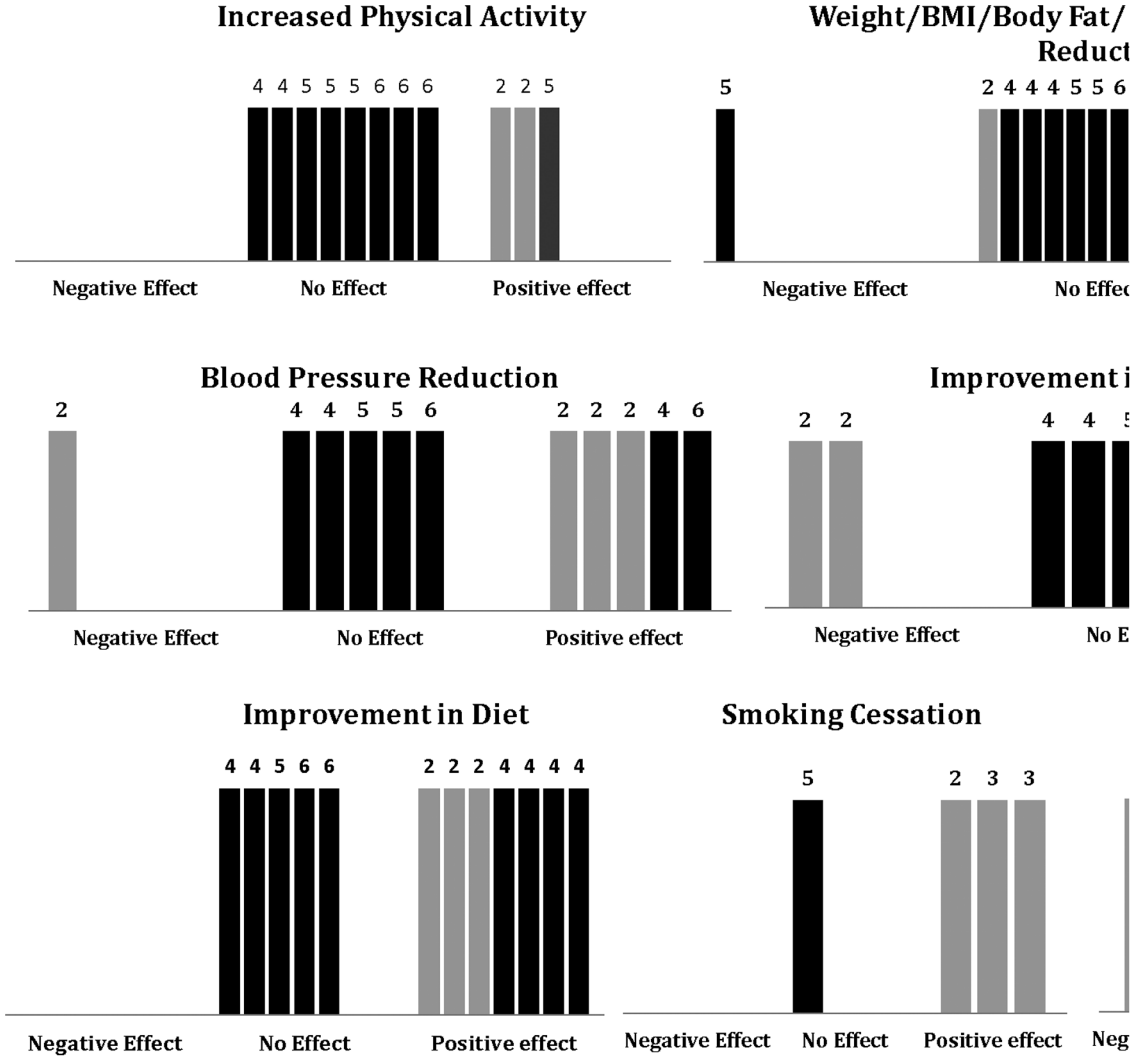
Graphical Representation of Intervention Outcomes (Modified from Harvest Plots by Ogilvie et. al). [11] Each bar represents a study. Dark bars indicate suitable study designs (category A or B) while the lighter bars indicate poor study design (category C, D or E). The numbers on top of each bar indicate the number of methodological criteria met (maximum 6). For each parameter, there are three possible outcomes – negative effect, no effect or positive effect.

The effects of the study interventions on the individual outcomes are graphically expressed using bar charts modified from Ogilvie et al. 's harvest plots[11] (figure 2). Each bar represents a study. The dark bars indicate adequacy of study design (A or B) while the lighter bars indicate inadequate study design (C, D or E). The numbers on each bar indicate the number of quality criteria met (maximum of 6). The modified harvest-plots are created for each outcome (figure 2).

Weight-related outcomes. Weight related outcomes were the most reported among the studies included in this review. In all, twenty studies reported on weight, BMI/obesity, waist circumference, skin fold thickness, and body fat changes of which 15 were high quality studies and 5 were of low-quality. Among the high quality studies, less than half (7) reported significant improvement in any weight related outcome[12], [14], [16], [22], [23], [26], [29]. The same number of high quality studies reported no significant change in the outcomes[15], [17], [20], [21], [24], [25], [27] while the last high quality study reported worsening of the outcome, in this case an increase in body fat[28]. One of the trials [15] with no significant intervention effect was a 2 year follow-up on a study [26] that showed a significant reduction in weight-related outcomes at 6 months.

Among the high quality studies reporting positive weight related outcomes, three noted significant reduction in weight[12], [23], [26]_ENREF_37, 5 reported reductions in waist circumference [14], [16], [23], [26], [29], while one reported significant reduction in body fat [22]. Six non-randomized studies reported weight related outcomes, five of which were of low-quality. Among the low-quality studies, (all non-randomized studies) only one did not find a significant improvement in weight or waist circumference. The reductions were modest and ranged between 0.8-1.4kg for weight [36], [38], and 2.0 – 2.9 cm for waist circumference [32], [33].

#### Blood pressure

Eleven studies reported BP as an outcome, 7 of which were high quality. Five of the 7 high quality studies did not find a significant effect of the intervention on blood pressure [14], [16], [23], [26], [27]while the other two reported clinically significant reduction in BP (reduction in SBP ≥10 mmHg or reduction in DBP ≥5 mmHg) [29], [30]. None of the high quality studies reporting no intervention effect on BP were conducted among at-risk populations (had elevated blood pressure).

All four pre-post studies reporting BP outcomes were of low-quality. Three of these reported a decline in BP[32], [33], [36], while one showed a paradoxical increase over the study period [35]. The latter was conducted in a group of overweight employees over a period of 24 months [35].

#### Blood glucose and HbA1c

Only one high quality study, a randomized trial, examined blood glucose profile as a study outcome[29]. This study, conducted among persons with type 2 diabetes mellitus or impaired fasting glucose over 12 and 24 months showed the internet-based intervention to be useful in reducing fasting plasma glucose (average of 12 mg/dl or 0.67 mmol/l less in the intervention group), while HbA1c was reduced only in those who received the intervention for two years [29].

The 2 pre-post studies (both low-quality) that reported blood or plasma glucose levels had contrasting results with one reporting a reduction in fasting glucose over 6 months, [32] while the other reported a significant increase in blood glucose (drawn at random) over 2 years [35].

#### Lipids

Six high-quality randomized studies reported on lipid profiles outcomes three of which showed improvements[18], [19], [29]. Among the other three high-quality studies reporting no significant intervention effect, 2 of them had follow-up periods of 2 years[15], [27].

Three pre-post studies (all low-quality) also assessed lipid outcomes. One demonstrated significant reduction in triglycerides but not HDL-c over 6 months[32]. Another [33] showed mixed results with an elevation in average total cholesterol and triglycerides (TG), while demonstrating an increase in HDL-c over the 7 month follow-up. The third study [35] demonstrated a reduction in HDL-c over 2 years (p = 0.033).

#### Diet

Nine high-quality studies, all randomized trials measured dietary outcomes, of which 5 found no improvements in any dietary outcome[16], [17], [23], [25], [27] while four demonstrated significant intervention effects on diet. Among those reporting improvements, the outcome measures were different. For instance, Bennet et al.[14] demonstrated improved dietary self-efficacy and healthy diet attitude, Papadaki et al.[18], [19] demonstrated greater intake in fruits, nuts, and seeds in the intervention group compared to controls at 6 months only, while Kang et al.[29] reported lower protein and sodium intake in the intervention group (administered over 2 years) compared to controls [14], [18], [19], [29].

Among the follow-up studies, 3 (all low-quality) demonstrated increases in fruit and vegetable intake [36]–[38]. One of them [36] also demonstrated a decrease in grain intake over 12 month follow-up (p<0.05). None showed a negative effect on dietary practices.

#### Physical Activity

Nine high quality randomized studies assessed physical activity as an outcome. Among these only one demonstrated a significant intervention effect on physical activity [20]. This study demonstrated that moderate exercise for ≥30 minutes/day on 5 or more days a week occurred 10% more in the intervention group compared to controls. In contrast, the other 9 high quality studies did not demonstrate a significant intervention effect [13], [16], [17], [21]–[23], [25], [26], [28].

The 2 low-quality studies that examined physical activity outcomes reported significant improvements[38], [40]. Both studies measured the increase in the number of people completing 10,000 steps per day with one of them combining the use of pedometers, website access, e-journaling, and social network approaches in their intervention [40].

#### Smoking cessation

Three follow-up studies that measured smoking cessation all showed significant intervention effects though they were all assessed to be of low-quality[31], [34], [39]. The study with the greatest intervention effect also had the highest intervention adherence rate [31]. In this study, 50% quit rate was achieved among University staff who participated in a multi-component cessation program. None of the randomized trials had a smoking cessation program although one trial measured smoking cessation as an outcome and found no significant difference in quit rates between the intervention and the control groups[20].

## Discussion

In general, the internet-based studies included in our review did not show consistent improvement in any of the outcomes assessed. Weight related and physical activity outcomes were the most examined and thus had the largest number of studies. Our findings show equal number of high quality studies reporting no improvement or some improvement on weight related changes however virtually all the high quality randomized trials showed no effect of the interventions on physical activity. Thus we may conclude that these types of interventions do not improve physical activity and unpredictable effects on weight management. Among the studies with dietary outcomes the number of high quality studies demonstrating improvements was similar to those with no significant intervention effect (4 vs. 5) making decisive conclusions about the efficacy of internet – based studies on improving diet difficult to make. More studies showed no effect on BP than significant BP reduction. However, we note that that only one study was targeted at persons who were hypertensive [30] and showed clinically significant reduction in blood pressure. Thus we conclude that general internet–based wellness interventions (multi-hit programs) may not be effective at BP reduction and that there is insufficient evidence to conclude for or against internet-based interventions targeted at persons with elevated BP/hypertension. Half of the six high quality studies examining lipid profiles showed no improvement while the other half demonstrated improvement in at least one parameter, thus making conclusions about internet based studies in improving lipid profiles impossible. There were too few high quality studies examining smoking cessation and improvement in blood glucose or HbA1c to comment on the effect of internet based interventions on these outcomes. This summary is made with caution since we have observed wide differences in intervention design, measured outcomes, populations studied and duration of follow-up in studies included in our review.

Comparing multi-component studies to those that were largely-internet based is difficult because of the differences in populations and intervention types. Still, one study compared 2 different delivery methods of the same intervention with no-intervention controls; a multi-component arm (in-person, telephone and email delivery), and an internet only program with similar content. This study found that the multi-component intervention group had significant improvements in physical activity, diet and waist circumference reduction compared to controls, while no improvements were seen in the internet arm compared to controls[16]. In several of the studies, having an environmental component to the intervention was more effective than having only web-based components. For instance, Watson et al. demonstrated clinically significant reduction in BP using a program that had internet-based reminders but also made BP measuring machines available in the workplace.

The impact of internet-based interventions in the long-term could not be assessed as most of the studies included in our review had relatively short follow-up periods with only a few lasting for one year or more. A look at trials with one or more years of follow-up (n = 7) showed that in over 70% (n = 5) of them no association was found between the interventions and improvements in any of the cardiovascular parameters measured. Two studies examining the same intervention at 6 months and 2 years showed association between the intervention and weight loss, waist circumference reduction and increased physical activity reported at 6 months but not 2 years [15], [26]. Moreover, in one of the 2 randomized trials reporting significant findings at one or more years of follow-up, the only improvement reported in the internet group compared to controls was a reduction in waist circumference[16]. A similar number of follow-up studies had follow-up periods of 12 months or more and they generally reported significant improvements in one or more of the outcomes. However, one study with a follow-up of 2 years reported a paradoxical increase in blood pressure, and worsening of the metabolic profile over the study period. The study was susceptible to areas of bias with regards to data collection and may not be a true reflection of the long term effect of internet based studies [35]. In general these findings may suggest that internet-based studies are minimally effective in the long term however there is not enough evidence to confirm this.

One of the AHA recommendations is to conduct more workplace cardiovascular wellness research involving high risk populations [3]. Not many high quality studies in our review were conducted in special risk groups – overweight/obese persons, those with diabetes mellitus/impaired fasting glucose, persons with hypertension, and populations with mean age of 50 years or more. Only 4 high quality studies (randomized trials) were conducted primarily on overweight/obese workers[17], [23], [25], [26] half of which did not show any significant improvement. Even fewer high-quality studies (two) were conducted in persons with a mean age of 50 years or more. Both of them showed reduction in waist circumference [16] and blood pressure [30]. The only randomized trial conducted in persons with diabetes or at risk for it showed significant improvement in fasting plasma glucose, HbA1c, lipids, diet, and reduction in systolic blood pressure [29]. Also as earlier mentioned only one study was conducted in those with hypertension and it demonstrated clinically significant reductions in both systolic and diastolic blood pressure [30]. More high–quality internet based intervention studies in special risk groups need to conducted to determine their utility and value in these populations.

Although we found no review on workplace internet-based CV wellness programs, several reviews on workplace wellness programs with CV outcomes have been conducted. Our findings on weight related outcomes were similar to those of Benedict & Arterburn (2008) who concluded that these programs have modest short term improvements in body weight [5]. A recent review found small intervention effect of workplace wellness programs on diet, noting that there was not enough evidence to comment on the long term effect of these interventions on dietary outcomes [7]. Similarly, we do not have enough evidence to comment on the long term effects of internet-based CV wellness programs on dietary outcomes and the available evidence is indeterminate of its short to intermediate effect.

A systematic review by Groeneveld et al. on workplace life-style focused interventions to reduce cardiovascular risk concluded that there was no effect of these programs on blood pressure, or on serum lipids [6]. They also had too little evidence to show an effect on blood glucose. Our findings on blood pressure are not entirely in agreement with Groeneveld et al [6]. Even though more high-quality studies reported no significant blood pressure changes, the two trials conducted in at-risk groups (those with hypertension or diabetes), demonstrated blood pressure reduction was universal and clinically significant [29], [30]. However, like Groeneveld et al., only few of our studies assessed blood glucose or HbA1c making it difficult to reach conclusions regarding the effect of internet-based interventions on these outcomes. Improvements in lipids profile were found in 3 of the 6 high-quality studies that examined these outcomes. Among these, 2 were reporting the same intervention at 6 and 9 month follow-up. On the other hand, 3 pre-post studies had improvement in the lipids profile while one showed a reduction in HDL-c [35]. As such, similar to Groeneveld et al. [6], we have limited evidence of an intervention effect on lipids profile.

A 2008 Cochrane review of smoking cessation programs in the workplace found strong evidence demonstrating that interventions directed at smokers were successful, and that interventions with multi-components including pharmacologic therapy were more successful in absolute terms than self-help and social support interventions [41]. The review noted that comprehensive programs were ineffective at promoting smoking cessation [41]. Although no randomized trials with smoking cessation interventions met our criteria, evidence from the follow-up studies support these findings.

Our review is limited in several ways. The decision to exclude studies with less than 6 months follow-up may have excluded well designed studies with short follow-up. However, in our opinion, for any intervention to have significant sustained effect a minimum follow-up time of 6 months is necessary. Some studies may have fit our inclusion criteria but did not show up in our search. To minimize this we examined the references of the publications found and those of other work place wellness reviews for relevant studies.

Due to the dissimilarity of the interventions studied, the heterogeneity of the outcomes and the disparate study design and quality, conducting a meta-analysis and interpreting its results would have been without merit. These problems along with differences in populations (varying age groups and gender distributions), cultural, and geographic regions where the studies were conducted have made it difficult to interpret the findings of the studies collectively. Finally, we cannot rule out the possibility of publication bias affecting our results.

### Implications and Conclusions

With rising costs of healthcare, many organizations have turned to employee health programs as a means of reducing disease burden, productivity losses and high management costs of illness. Although there is controversy and skepticism as to the clinical utility and cost-effectiveness of these programs [42], one study indicates that employee wellness programs offer an average of 3.37 USD reductions in medical costs for every dollar spent [43]. That said, focusing on health in the workplace could only be cost-effective if the interventions are both effective and cost saving.

The success of any intervention depends on many factors including the method of delivery. Several reviews have suggested that internet delivered programs may be effective, albeit marginal at improving health behavior [44], [45], managing weight [46], [47], or increasing physical activity [48], [49], and at least one review suggested that internet-based programs may be more effective at promoting healthy behaviors than non-internet-based interventions [45]. However, none of these reviews were conducted in primarily working populations neither did they evaluate other outcomes such as BP reduction and lipid profile changes, all of which this study does.

Our review highlights the need for internet-based workplace prevention and wellness programs to include some physical contact with participants and environmental modification to improve effectiveness however knowing the right blend of internet and personal contact components for each intervention also poses significant challenge. For desired outcomes such as smoking cessation and blood pressure reduction, interventions perhaps should be tailored to the specific needs of each working population. High risk populations – persons with one or more significant risk for CVD - make up a significant proportion of the working population and are a major group that is recommended for workplace wellness programs. Unfortunately, most studies were conducted in healthy populations and as such the most effective means of delivering CV wellness programs to these populations are still unclear. Finally, for most of the reviewed programs, formulating a clear picture on their long term implications awaits further studies with longer follow-up.

Although this review demonstrates potential for success of internet-based programs at reducing cardiovascular disease morbidity as seen in weight reduction, blood pressure control and smoking cessation, much work needs to be done to understand the best approaches involving internet use in delivery of cardiovascular wellness programs and their long term effectiveness.

Adopting some standardized outcomes such as those suggested in the AHA 2020 guidelines will further help synthesize the totality of evidence about the effectiveness of these programs and provide the much needed evidence for healthcare workers, policy makers and the business community to make meaningful decisions as to the most effective ways of employing the internet in the delivery of CV wellness programs.

## Supporting Information

Table S1 **Methodological Criteria as Described by Ogilvie et al.**
(DOCX)

Table S2 **Table Demonstrating Methodological Scoring for individual studies using the Ogilvie et al. criteria.**
(DOCX)

Checklist S1 **PRISMA Checklist.**
(DOCX)
