# Correction: A Systematic Review of Internet-Based Worksite Wellness Approaches for Cardiovascular Disease Risk Management: Outcomes, Challenges & Opportunities

**DOI:** 10.1371/journal.pone.0092759

**Published:** 2014-03-14

**Authors:** The PLOS ONE Staff

There is an error in Figure 2. Please see the correct Figure 2 here:

**Figure 2:**
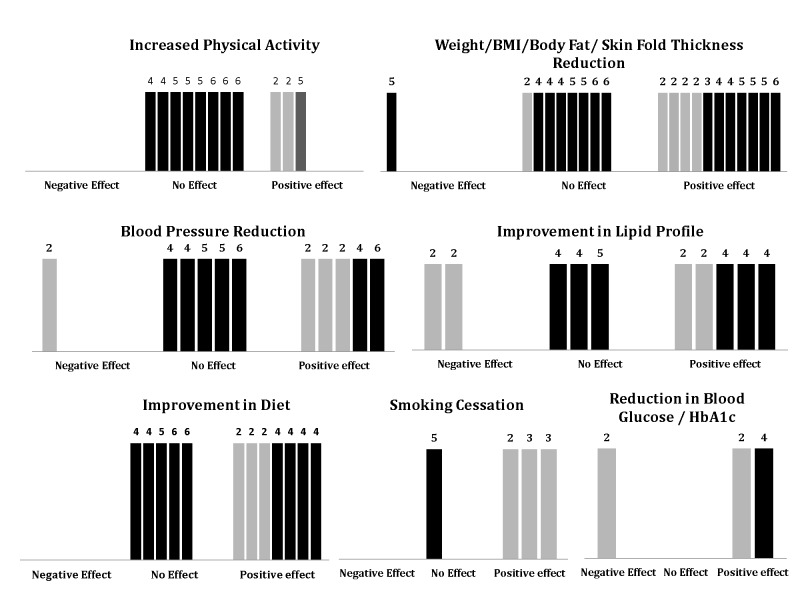
Graphical Representation of Intervention Outcomes (Modified from Harvest Plots by Ogilvie et. al). [11] Each bar represents a study. Dark bars indicate suitable study designs (category A or B) while the lighter bars indicate poor study design (category C, D or E). The numbers on top of each bar indicate the number of methodological criteria met (maximum 6). For each parameter, there are three possible outcomes – negative effect, no effect or positive effect.
