# Supplementary material for: A Systematic Review of Internet-Based Worksite Wellness Approaches for Cardiovascular Disease Risk Management: Outcomes, Challenges & Opportunities
Source: PLoS One. 2014 Jan 8;9(1):e83594. doi: 10.1371/journal.pone.0083594 (PMC3885454; doi:10.1371/journal.pone.0083594)
Supplement: Table S2 — Table Demonstrating Methodological Scoring for individual studies using the Ogilvie et al. criteria. (DOCX) [file pone.0083594.s002.docx]

| **Table S2a - Summary Table Demonstrating Methodological Scoring Using the Ogilvie et al. criteria: Randomized Studies** | | | | | | |
| --- | --- | --- | --- | --- | --- | --- |
| **Study name (first author)** | Tate | Spittaels | Morgan | Bennet | Watson | Papadaki 2008 |
| **Category** | A | A | A | A | A | A |
| **Representativeness** | Y | Y | Y | N | Y | Y |
| **Randomization** | Y | Y | Y | Y | Y | N |
| **Comparability** | Y | Y | Y | Y | Y | N |
| **Credibility** | Y | N | Y | Y | Y | Y |
| **Attrition** | Y | Y | Y | N | Y | Y |
| **Attributability** | Y | Y | Y | Y | Y | Y |
| **Score** | A, 6/6 | A, 5/6 | A, 6/6 | A, 4/6 | A, 6/6 | A, 4/6 |
|  | | | | | | |
| **Study name**  **(first author)** | Papadaki | Prochaska | Van Wier | Van Genugten | Slootmaker | Hughes |
| **Category** | A | A | A | A | A | A |
| **Representativeness** | Y | N | N | Y | Y | Y |
| **Randomization** | N | Y | Y | Y | Y | Y |
| **Comparability** | N | Y | Y | N | Y | N |
| **Credibility** | Y | Y | Y | Y | Y | Y |
| **Attrition** | Y | Y | Y | N | Y | Y |
| **Attributability** | Y | Y | Y | Y | Y | Y |
| **Score** | A, 4/6 | A, 5/6 | A,5/6 | A, 4/6 | A 6/6 | A, 5/6 |
|  | | | | | | |
| **Study name**  **(first author)** | Thorndike | Aittasalo | Reijonsaari | Kang | Dekkers | Robroek |
| **Category** | A | A | A | A | A | A |
| **Representativeness** | N | N | N | N | N | N |
| **Randomization** | Y | Y | Y | Y | Y | Y |
| **Comparability** | Y | Y | Y | N | Y | Y |
| **Credibility** | Y | Y | Y | Y | Y | Y |
| **Attrition** | Y | Y | Y | Y | N | N |
| **Attributability** | Y | Y | Y | Y | Y | Y |
| **Score** | A 5/6 | A 5/6 | A 5/6 | A 4/6 | A 4/6 | A 4/6 |

| **Table S2b - Summary Table Demonstrating Methodological Scoring Using the Ogilvie et al. criteria: Non-randomized Studies** | | | | | | |
| --- | --- | --- | --- | --- | --- | --- |
| **Study name (first author)** | Jung | Speck | Pratt | Colkessen | Moore | Perez |
| **Category** | C | C | C | C | C | C |
| **Representativeness** | N | N | N | N | N | N |
| **Randomization** | N | N | N | N | N | N |
| **Comparability** | N | N | N | N | N | N |
| **Credibility** | Y | Y | Y | Y | Y | Y |
| **Attrition** | N | N | N | N | N | N |
| **Attributability** | Y | Y | Y | Y | Y | Y |
| **Score** | C2/6 | C, 2/6 | C, 2/6 | C, 2/6 | C, 2/6 | C, 2/6 |
|  | | | | | | |
| **Study name**  **(first author)** | Peterson | Hotta | Graham | Sarna | McHugh |  |
| **Category** | A | C | C | C | C |  |
| **Representativeness** | N | N | N | N | N |  |
| **Randomization** | N | N | N | N | N |  |
| **Comparability** | Y | N | N | N | N |  |
| **Credibility** | Y | Y | Y | Y | Y |  |
| **Attrition** | N | Y | Y | N | N |  |
| **Attributability** | Y | Y | Y | Y | Y |  |
| **Score** | A, 3/6 | C, 3/6 | C, 3/6 | C, 2/6 | C, 2/6 |  |
